# Metabolic reprogramming and lipid droplets are involved in Zika virus replication in neural cells

**DOI:** 10.1186/s12974-023-02736-7

**Published:** 2023-03-08

**Authors:** Suelen Silva Gomes Dias, Tamires Cunha-Fernandes, Luciana Souza-Moreira, Vinicius Cardoso Soares, Giselle Barbosa Lima, Isaclaudia G. Azevedo-Quintanilha, Julia Santos, Filipe Pereira-Dutra, Caroline Freitas, Patricia A. Reis, Stevens Kastrup Rehen, Fernando A. Bozza, Thiago M. Lopes Souza, Cecilia J. G. de Almeida, Patricia T. Bozza

**Affiliations:** 1 Laboratório de Imunofarmacologia, Instituto Oswaldo Cruz (IOC), Fundação Oswaldo Cruz (FIOCRUZ), Rio de Janeiro, Brazil; 2 Programa de Imunologia e Inflamação, Universidade Federal do Rio de Janeiro, UFRJ, Rio de Janeiro Rio de Janeiro, Brazil; 3 Departamento de Bioquímica, Instituto de Biologia Roberto Alcântara Gomes, Universidade Estadual do Rio de Janeiro, Rio de Janeiro, Brazil; 4 Instituto D’Or de Pesquisa e Ensino (IDOR), Rio de Janeiro, Brazil; 5 Instituto de Biologia, Universidade Federal do Rio de Janeiro, UFRJ, Rio de Janeiro, Rio de Janeiro Brazil; 6 Instituto Nacional de Infectologia Evandro Chagas (INI), FIOCRUZ, Rio de Janeiro, Brazil; 7 Instituto Nacional de Ciência e Tecnologia em Inovação em Doenças de Populações Negligenciadas (INCT/IDPN), Centro de Desenvolvimento Tecnológico em Saúde, (CDTS), FIOCRUZ, Rio de Janeiro, Brazil

**Keywords:** Lipid metabolism, Lipid droplets, Immunometabolism, Inflammation, Zika virus, Neuroinfection

## Abstract

Zika virus (ZIKV) infection is a global public health concern linked to adult neurological disorders and congenital diseases in newborns. Host lipid metabolism, including lipid droplet (LD) biogenesis, has been associated with viral replication and pathogenesis of different viruses. However, the mechanisms of LD formation and their roles in ZIKV infection in neural cells are still unclear. Here, we demonstrate that ZIKV regulates the expression of pathways associated with lipid metabolism, including the upregulation and activation of lipogenesis-associated transcription factors and decreased expression of lipolysis-associated proteins, leading to significant LD accumulation in human neuroblastoma SH-SY5Y cells and in neural stem cells (NSCs). Pharmacological inhibition of DGAT-1 decreased LD accumulation and ZIKV replication in vitro in human cells and in an in vivo mouse model of infection. In accordance with the role of LDs in the regulation of inflammation and innate immunity, we show that blocking LD formation has major roles in inflammatory cytokine production in the brain. Moreover, we observed that inhibition of DGAT-1 inhibited the weight loss and mortality induced by ZIKV infection in vivo. Our results reveal that LD biogenesis triggered by ZIKV infection is a crucial step for ZIKV replication and pathogenesis in neural cells. Therefore, targeting lipid metabolism and LD biogenesis may represent potential strategies for anti-ZIKV treatment development.

**Supplementary Information:**

The online version contains supplementary material available at 10.1186/s12974-023-02736-7.

## Introduction

The outbreak of ZIKV infection caused great alarm worldwide, as the relationship between infection with ZIKV and cases of microcephaly in neonates was demonstrated [1, 2] and associated with Guillain–Barré Syndrome in adults [3, 4]. ZIKV shows a remarkable tropism for neural cells and prevents brain development, leading to irreversible damage [5–7]. ZIKV is an arbovirus (arthropod-borne virus) and belongs to the family *Flaviviridae*, genus *Flavivirus* [8]. Its genome is a single-stranded ribonucleic acid (RNA) with positive-sense (+ RNA) and encodes a single polyprotein that undergoes cleavage generating three structural and seven nonstructural proteins [9–11].

During many stages of the viral replication cycle in the cytoplasm, + RNA viruses interact with host proteins and alter cell homeostasis to benefit viral replication and assembly [12, 13]. Members of the family Flaviviridae manipulate host lipid metabolism and induce lipid droplet (LD) biogenesis, as observed in Dengue virus (DENV)- [14] and Hepatitis C virus (HCV)-infected cells [15]. LDs are dynamic organelles consisting of a core rich in neutral lipids surrounded by a phospholipid monolayer and structural proteins of the Perilipin family (PLIN1-5) [16, 17]. LDs play an essential role in cellular lipid storage and homeostasis [18], intracellular transport, and inflammatory processes [19]. Moreover, different pathogens, including bacteria, viruses, and parasite infection, demonstrate the ability to modulate the lipid metabolism of the host cell, favoring its survival and replication [20–22]. In this context, multiple + RNA viruses use the host lipid machinery to facilitate their replication and assembly [23].

Pharmacological interference in lipid metabolism and LD inhibition affects viral replication of different viruses. Inhibition of fatty acid synthase (FASN) or acyl-CoA:diacylglycerol acyltransferase-1 (DGAT-1) impairs the replication of HCV, DENV and SARS-CoV-2 [14, 24, 25]. DGAT-1 is a key endoplasmic membrane-bound enzyme for triacylglycerol (TAG) synthesis and LD formation. Therefore, these data demonstrate the essential role of lipid metabolism and LDs during + RNA viral replication. However, the role of LDs during ZIKV infection is still poorly explored.

Recent studies have suggested functions for LDs in the central nervous system [26]. Indeed, cells of the nervous system exhibit LDs in the context of development, obesity, and neurodegenerative pathologies [26–28], but to our knowledge, no studies have addressed the functions of CNS LDs in the context of infection.

We hypothesize that LDs may contribute to ZIKV infection-induced neuropathology. Here, we demonstrate that ZIKV infection alters cellular lipid metabolism in human neural cells, decreasing lipogenesis and increasing de novo lipid synthesis and remodeling. As a result, LD organelles accumulate in the cytoplasm, providing a favorable environment for viral replication. In addition, preventing LD biogenesis with a pharmacological inhibitor of DGAT-1 impairs viral replication in vitro and in vivo. Accordingly, the DGAT-1 inhibitor decreases inflammatory cytokine production, weight loss and mouse mortality induced by ZIKV infection. Our results demonstrate that ZIKV virus infection alters host cell lipid metabolism to benefit ZIKV replication and may impact the development of therapies to combat ZIKV infection.

## Results

### ZIKV infection alters lipid metabolism in human neuroblastoma cells

**Fig. 1:**
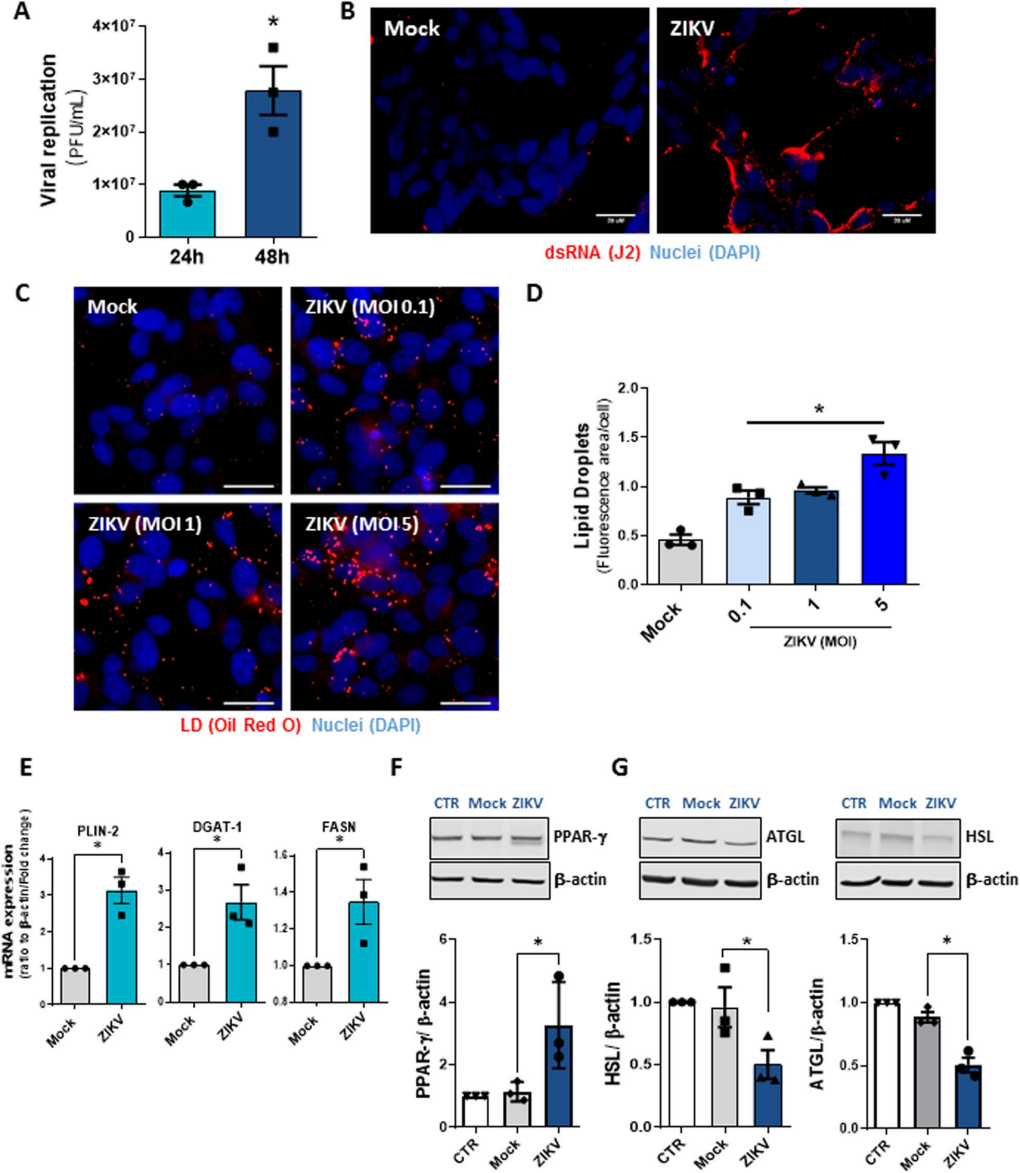
ZIKV infection alters lipid metabolism in human neuroblastoma cells. **A** Viral replication was analyzed by PFU assay in SH-SY5Y cells after 24 h and 48 h of ZIKV infection at an MOI of 1. **B** Immunofluorescence analyses of SH-SY5Y cells after ZIKV infection at an MOI of 1 for 48 h. The double strain RNA was detected by indirect immunofluorescence with a J2 antibody (red), and the nuclei were stained with DAPI (blue). The scale bar represents 20 µm in range. **C** Images of SH-SY5Y cells infected with ZIKV at MOIs of 0.1, 1, and 5 after 48 h. The cells were stained with Oil Red O (Red) and DAPI (blue) for nuclei. The scale bar represents 10 µm in range. **D** Quantification of the fluorescence area per cell in SH-SY5Y cells. **E** Real-time PCR for proteins related to lipid metabolism after 24 h of ZIKV infection. **F** Representative western blot analysis and densitometry data set of PPAR-gamma (**G**) and the lipolytic enzymes ATGL and HSL in SH-SY5Y lysates 48 h post ZIKV infection. Data information: In **A**, **D**–**F**, data are presented as the means ± SEMs of three independent experiments. *P < 0.05 mock- versus ZIKV-infected cells

*Flaviviridae* members strictly depend on host resources and induce metabolic alterations, modulating lipid metabolism to support viral replication and assembly [29–32]. Here, we first confirmed the ability of neuroblastoma SH-SY5Y cells to produce ZIKV infectious particles. This cell line has been previously described as a model to study the infection of neural cells by different viruses [33–36], including ZIKV [37, 38]. SH-SY5Y cells were infected with African ZIKV strain MR766 for 24 h and 48 h at a multiplicity of infection (MOI) of 1. As shown in Fig. 1A, there was an increase in viral replication after infection in a time-dependent manner. Similar to other + RNA viruses, ZIKV produces double-stranded RNA (dsRNA) as an intermediate during viral replication into host cells. Thus, we used an antibody against dsRNA to confirm ZIKV infection and replication. At 48 h post-infection (hpi), SH-SY5Y-infected cells showed increased expression of dsRNA compared to uninfected cells (Mock) (Fig. 1B).

Several viruses trigger LD formation, which regulates host metabolism and lipid storage. This organelle supports viral replication and is central in the pathogenesis of various infections [14, 39, 40]. Indeed, ZIKV infection increased the biogenesis of LDs in SH-SY5Y cells in an MOI-dependent manner (Fig. 1C, D). To further gain insights into the mechanisms involved in LD formation induced by ZIKV infection in neuroblastoma cells, we analyzed the expression of regulatory factors associated with lipid metabolism. Interestingly, compared with uninfected cells, ZIKV-infected cells exhibited upregulated gene expression of PLIN-2, DGAT-1, and FASN (Fig. 1E). Additionally, SH-SY5Y cells infected with ZIKV showed increased expression of the lipogenesis transcription factor peroxisome proliferator-activated receptor-γ (PPAR-γ) (Fig. 1F). In contrast, significantly decreased expression of two lipolysis enzymes, adipose triglyceride lipase (ATGL) and hormone-sensitive lipase (HSL), was observed 48 h after infection (Fig. 1G).

### ZIKV infection modulates lipid metabolism in human neural stem cells

**Fig. 2:**
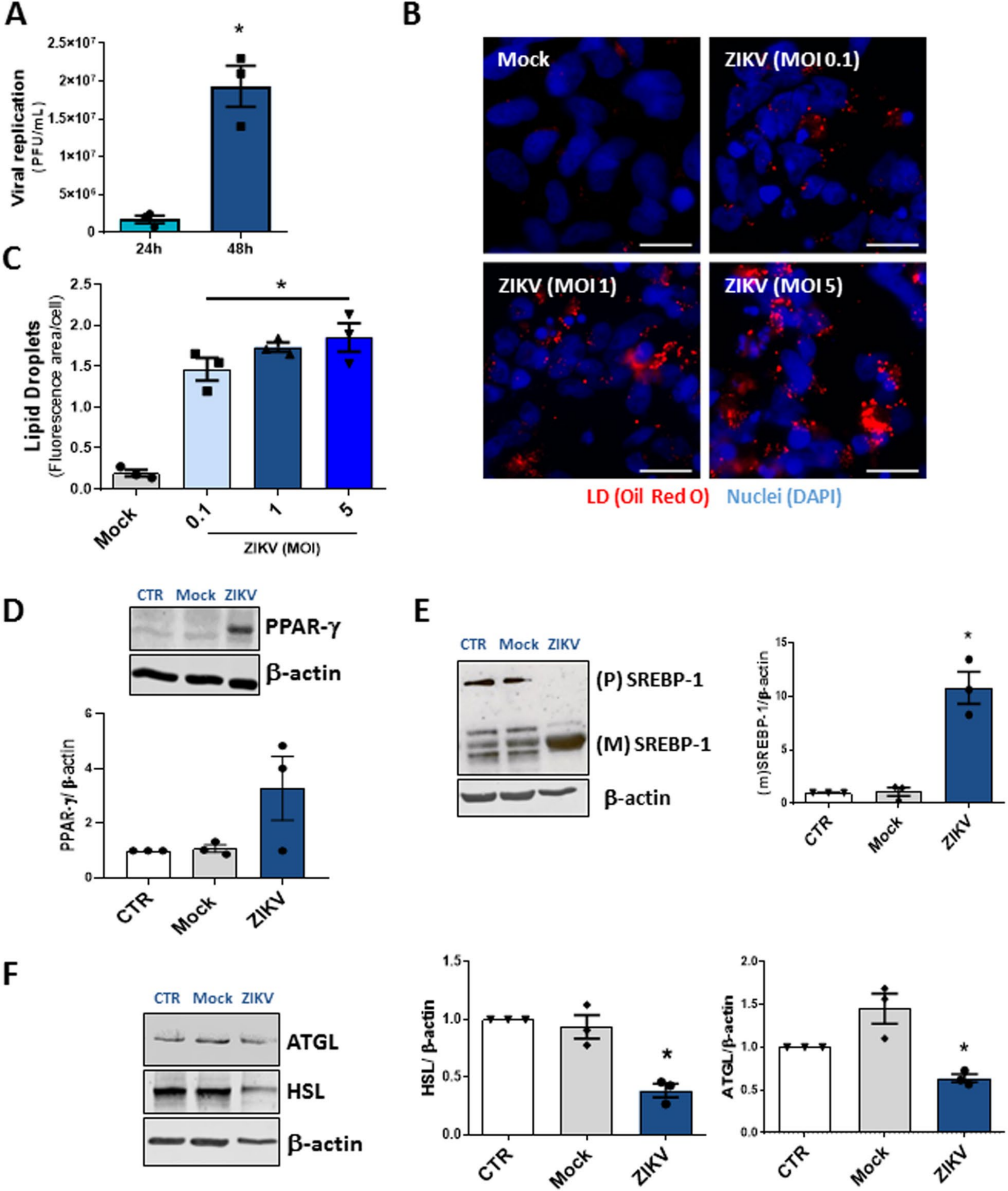
ZIKV infection modulates lipid metabolism in human neural stem cells. **A** Viral replication was analyzed by PFU assay in NSCs after 24 h and 48 h of ZIKV infection at an MOI of 1. **B** Images of NSCs infected with ZIKV at MOIs of 0.1, 1, and 5 after 48 h. The cells were stained with Oil Red O (Red) and DAPI (blue) for nuclei. The scale bar represents 10 µm in range. **C** Quantification of the fluorescence area per cell in NSCs. **D** Representative western blot analysis of PPAR-gamma, (**E**) precursor and mature SREBP-1, (**F**) ATGL and HSL in NSC lysates 48 h post-ZIKV infection at an MOI of 1. Data information: In A, C-D, data are presented as the means ± SEMs of three independent experiments. *P < 0.05 mock- versus ZIKV-infected cells

Next, we asked whether ZIKV infection alters lipid metabolism proteins and increases LD accumulation in NSCs derived from iPSCs. We observed that the production of ZIKV infective particles increased in a time-dependent manner in NSCs, as shown in Fig. 2A. Similar to the results with SH-SY5Y cells, ZIKV infection in NSCs induced the expressive accumulation of LDs compared with mock-treated NSCs (Fig. 2B, C).

Furthermore, ZIKV infection showed a trend to increase the expression of PPAR-γ compared with the uninfected cells CTR and Mock-treated NSCs (Fig. 2D). ZIKV infection reduced the precursor form of sterol regulatory element-binding protein 1 (SREBP-1) while significantly increasing the mature/active form of SREBP-1 at 48 hpi compared to that in uninfected cells (Fig. 2E). As observed in the neuroblastoma cell line, ZIKV infection in NSCs decreased the expression of ATGL and HSL compared with uninfected cells (Fig. 2F).

These data suggest that modulation of host lipid metabolism is critical during ZIKV infection in neuroblastoma cells and NSCs, as shown by the effects of increased lipogenesis and decreased lipolysis regulatory factors in neural cells favoring lipogenesis and LD accumulation, occurring in parallel with virus replication.

### Inhibition of LD accumulation decreases ZIKV replication

**Fig. 3:**
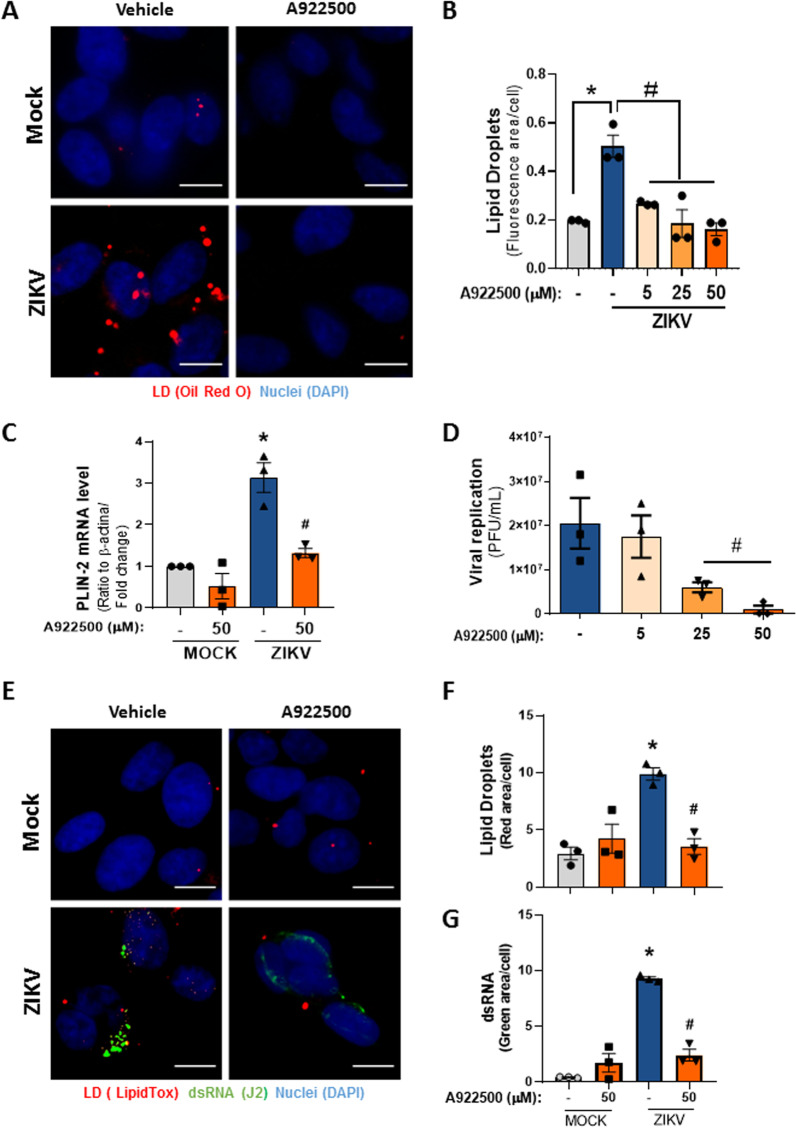
Inhibition of lipid droplet accumulation induced by ZIKV decreases viral replication in neuroblastoma cells. **A** Representative images of SH-SY5Y cells 48 h after ZIKV infection treated with 50 µM DGAT-1 inhibitor (A922500) and stained with Oil Red O (red) and DAPI (blue) for nuclei. The scale bar represents 10 µm in range. **B** Quantification of the fluorescent area per cell in each group. **C** Real-time PCR for PLIN-2 after 24 h of ZIKV infection and treatment with a DGAT-1 inhibitor. **D** Effect of the DGAT-1 inhibitor on ZIKV replication at 48 hpi. **E** Images of SH-SY5Y cells after 48 h of ZIKV infection and the ds-RNA stained with J2 antibody (green), the lipid droplets were stained with LipidTox (Red) and nuclei with DAPI (blue). The scale bar represents 10 µm in range. **F** Quantification of LD pixels per cell. **G** Quantification of ds-RNA pixels per cell. Data information: In **B**–**D**, **F**, **G**), data are presented as the means ± SEMs of three independent experiments. *P < 0.05 mock- versus ZIKV-infected cells. #*P* < 0.05 ZIKV-infected cells versus A922500 treatments

Pharmacological inhibition of enzymes involved in lipid metabolism and LD biogenesis impacts viral replication, as observed in DENV [14], HCV [41], rotavirus [42] and SARS-CoV-2 [25] infection models. To advance the understanding of the role of lipid accumulation during ZIKV infection in neural cells, we analyzed the pharmacological inhibition of the DGAT-1 enzyme, which catalyzes the terminal step in TAG synthesis. Compared with vehicle treatment, treatment with a DGAT-1 inhibitor (iDGAT-1, A922500) markedly decreased LD accumulation triggered by ZIKV infection in a concentration-dependent manner in SH-SY5Y cells (Fig. 3A, B; Additional file 1: Fig. S1A).

Corroborating these data, treatment with iDGAT-1 decreased the mRNA expression of PLIN-2, a structural LD protein induced by ZIKV infection (Fig. 3C). Moreover, iDGAT-1 treatment decreased ZIKV replication in SH-SY5Y cells (Fig. 3D). This observation is supported by the reduction in dsRNA labeling and LD accumulation observed by J2 clone antibody and LipidTox staining, respectively, after iDGAT-1 treatment (Fig. 3E). Further image analysis and quantification of dsRNA and LDs confirmed that treatment with iDGAT-1 significantly reduced LD production (Fig. 3F) and ZIKV replication (Fig. 3G).

**Fig. 4:**
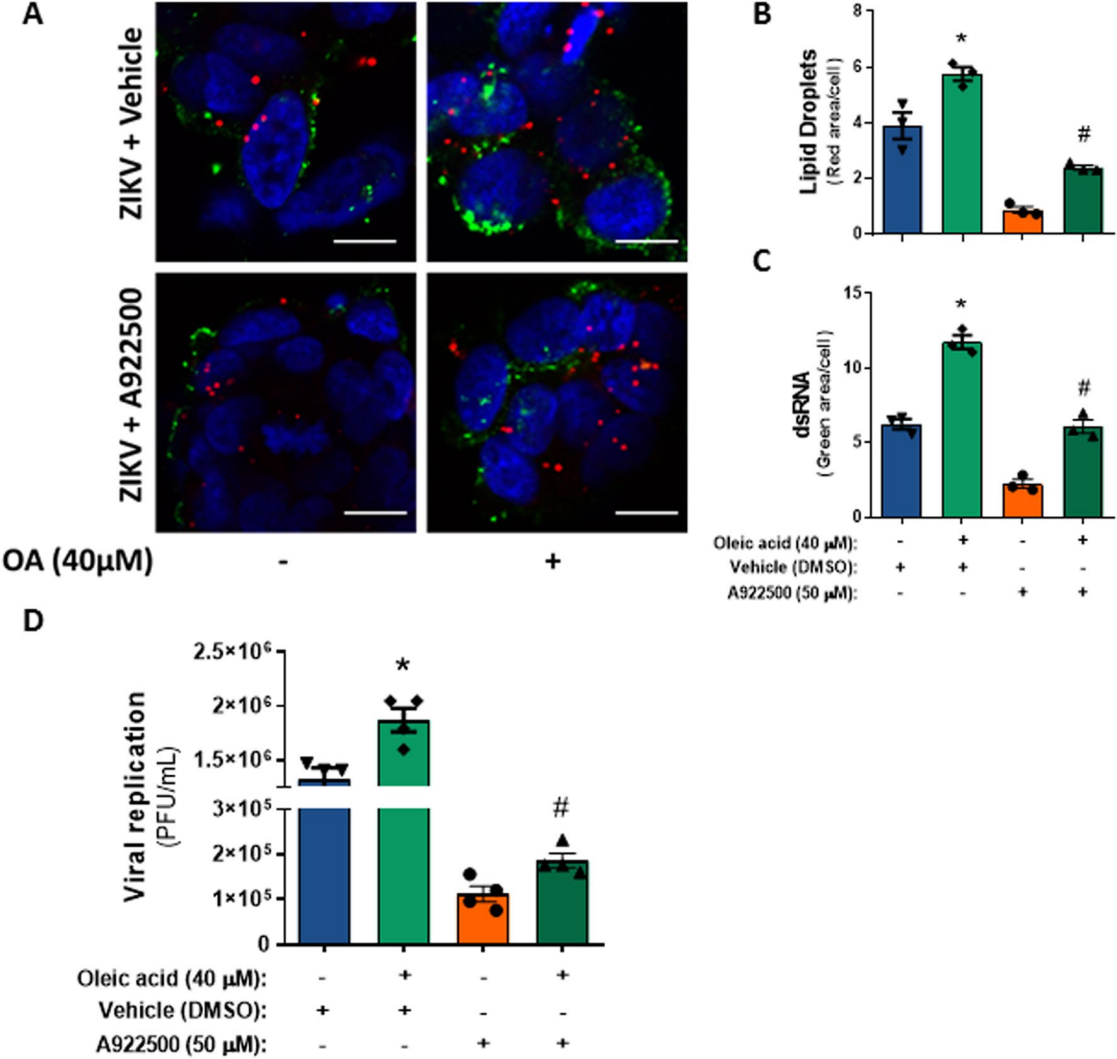
Upregulation of LD biogenesis increased ZIKV replication in SH-SY5Y cells. **A** Representative images of SH-SY5Y cells treated with 40 µM oleic acid and 50 µM DGAT-1 inhibitor (A922500) 48 h after ZIKV infection. The ds-RNA was stained with a J2 antibody (green), the lipid droplets were stained with LipidTox (red), and the nuclei were stained with DAPI (blue). The scale bar represents 10 µm in range. **B** Quantification of LD pixels per cell. **C** Quantification of ds-RNA pixels per cell. **D** Effect of the DGAT-1 inhibitor on ZIKV replication at 48 hpi. Data information: In **A**–**C**, data are presented as the means ± SEMs of three independent experiments, and in **D**, data are presented as the means ± SEMs of four independent experiments. *P < 0.05 Veh alone versus Veh with oleic acid-treated cells. #*P* < 0.05 A922500 alone versus A922500 with oleic acid-treated cells

Moreover, we evaluated whether the upregulation of LDs could affect ZIKV replication. SH-SY5Y cells were treated with oleic acid (40 µM) 1 h prior to ZIKV infection at an MOI of 1 and maintained after 48 h of infection. Interestingly, supplementation with 40 µM oleic acid increased LD accumulation and dsRNA labeling, as observed by LipidTox staining and J2 clone antibody, respectively (Fig. 4A–C, and ZIKV replication (Fig. 4D). To evaluate whether oleic acid treatment could rescue ZIKV replication during DGAT-1 inhibition, the cells were treated with oleic acid in the presence of a DGAT-1 inhibitor or vehicle during 48 h of ZIKV infection. We observed that oleic acid treatment was able to partially restore the inhibitory effect of DGAT-1 inhibition on LD accumulation and ZIKV replication (Fig. 4).

**Fig. 5:**
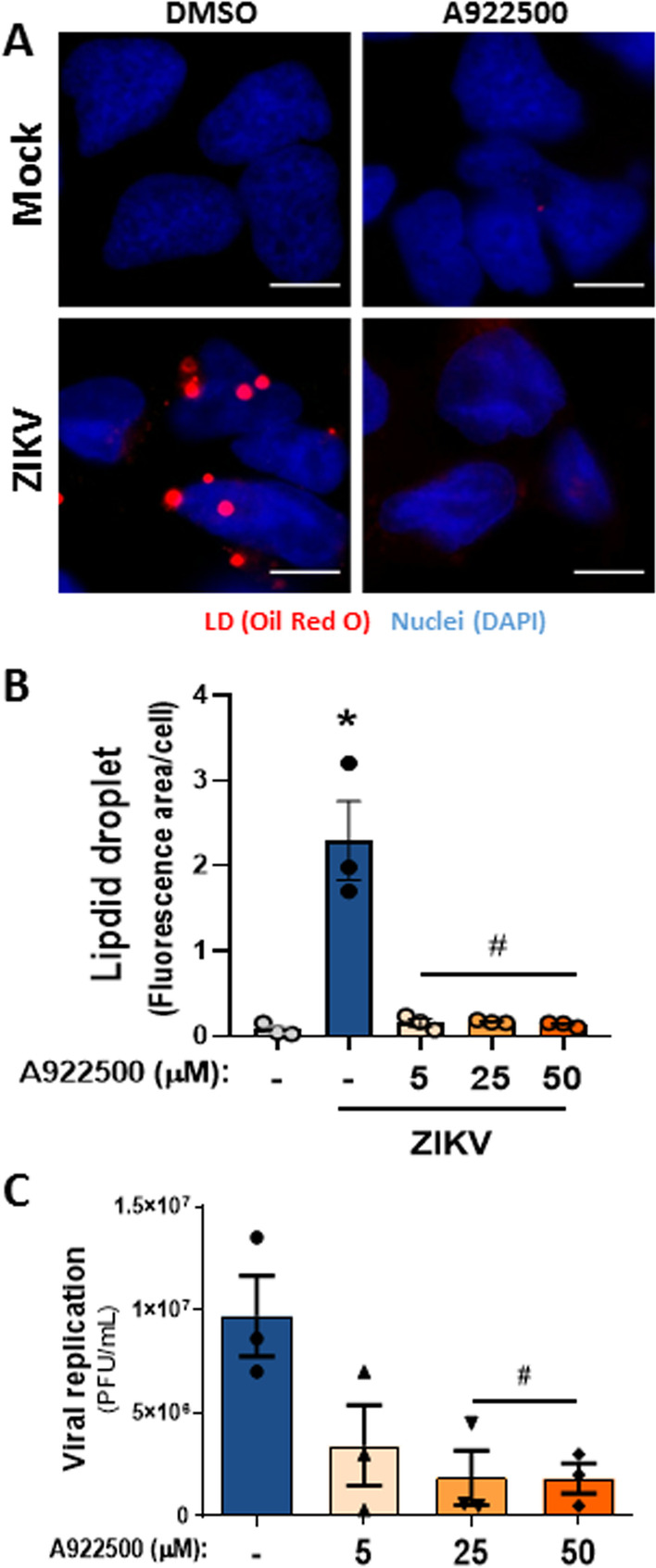
Effect of iDGAT-1 treatment on lipid droplet biogenesis and ZIKV replication in human neural stem cells. **A** Representative images of NSCs 48 h after ZIKV infection at an MOI of 1 treated with 50 µM DGAT-1 inhibitor (A922500) and stained with Oil Red O (red) and DAPI (blue) for nuclei. The scale bar represents 10 µm in range. **B** Quantification of the fluorescent area per cell in each group. **C** Effect of the DGAT-1 inhibitor on ZIKV replication at 48 hpi in NSCs. Data information: In **B**, **C**, data are presented as the means ± SEMs of three independent experiments. *P < 0.05 mock- versus ZIKV-infected cells. #*P* < 0.05 ZIKV-infected cells versus A922500 treatments

Furthermore, treatment with iDGAT-1 expressively reduced LD accumulation by ZIKV in NSCs, even when a lower concentration was used (Fig. 5A, B. Along with the observation in SH-SY5Y cells, treatment with iDGAT-1 significantly reduced ZIKV replication at 48 hpi in NSCs (Fig. 5C). Additionally, A922500 treatment alone did not affect the viability of uninfected cells, with a 50% cytotoxic concentration (CC_50_) value of 88.14 μM for SH-SY5Y cells and higher than 250 μM for NSCs (Additional file 2: Fig S2).

These findings suggest that TAG metabolism and LD accumulation may play a crucial role during the ZIKV cycle, as the inhibition of DGAT decreases LD accumulation and significantly impacts virus replication in both SH-SY5Y cells and NSCs.

### DGAT-1 treatment improves the survival of ZIKV-infected mice

**Fig. 6:**
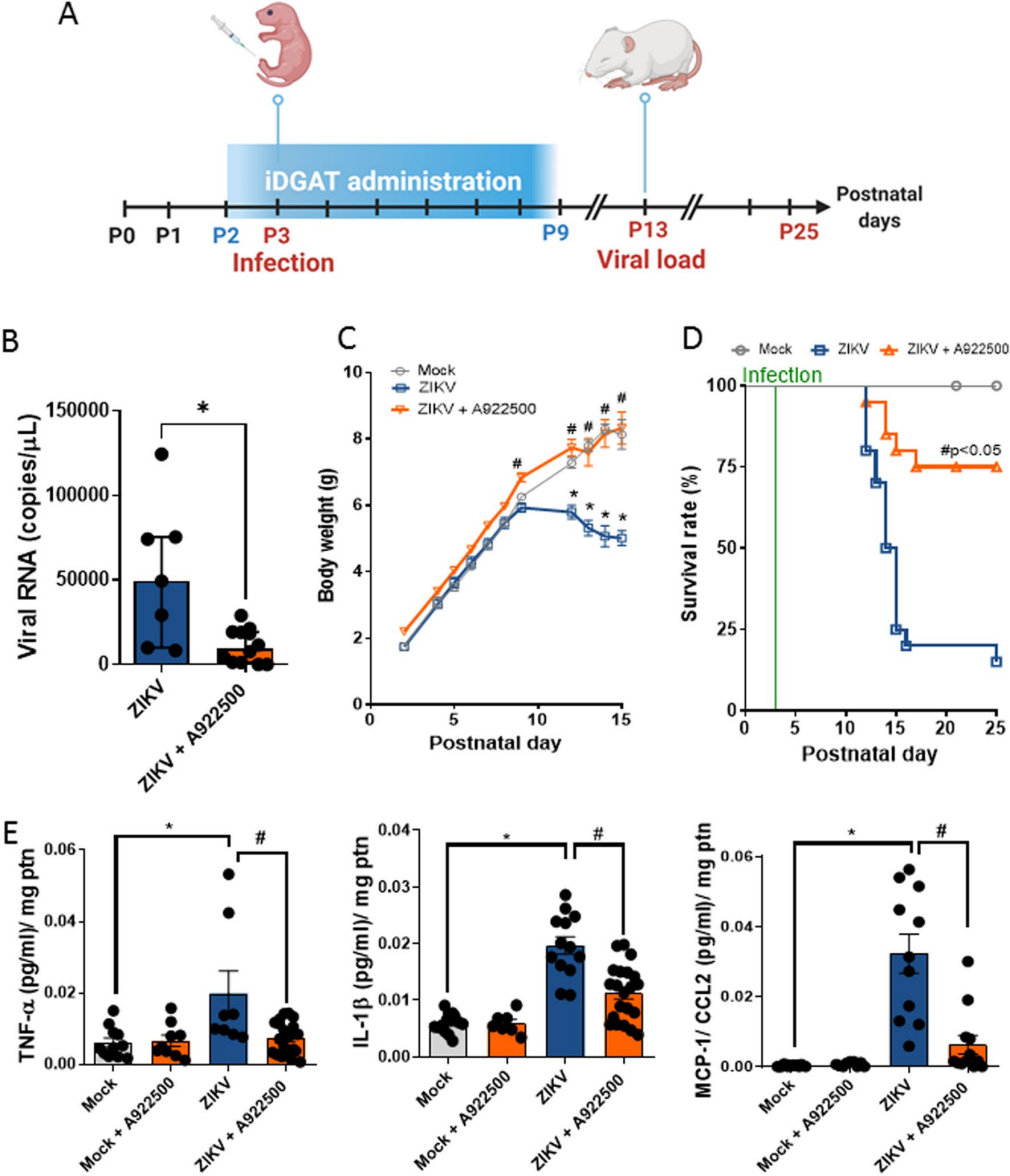
Treatment with DGAT-1 inhibitor (A922500, iDGAT) reduces viral loads in the brain during acute infection, increases survival, and inhibits weight loss in ZIKV-infected mice. **A** Three-day-old Swiss mice were infected with Brazilian ZIKV (2 × 10^4^ PFU) and treated with iDGAT-1 one day before infection for 7 days. On the indicated days after infection, animals were euthanized. **B** ZIKV RNA levels were measured in the brain. **C** Weight variation (**D**) and survival were assessed during treatment. **E** Inflammatory mediators were measured in mouse brain extracts by ELISA: TNF, IL-1β and MCP-1. Data information: In **B**, the viral loads are displayed as the mean ± SEM of seven ZIKV-infected mice, and twelve ZIKV-infected mice per day were assayed. Student's *t* test was used to compare the viral levels from ZIKV-infected vs. ZIKV-treated mice. *p < 0.05. In **C**, differences in weight are displayed as the mean ± SEM, and two-way ANOVA for each day was used to assess the significance. In **D**, survival was statistically assessed by the log-rank (Mantel‒Cox) test. Independent experiments were performed with ten mice/group (*n* = 30). *P < 0.05. In **E**, the inflammatory mediators are presented as the mean ± SEM of 14 mock mice, nine mock-treated, 13 ZIKV-infected mice and 27 ZIKV-treated mice. *P < 0.05 mock- versus ZIKV-infected mice. #*P* < 0.05 ZIKV-infected mice versus A922500 treatment

To further demonstrate the relevant role of TAG accumulation during ZIKV infection, we established an acute in vivo ZIKV infection model. We treated newborn (P2) to P9 mice with an intraperitoneal injection of the A922500 DGAT-1 inhibitor daily and infected them afterwards with a dose of 2 × 10^7^ PFU of Brazilian ZIKV strain to induce an acute infection according to previous studies [43, 44] (Fig. 6A).

Since iDGAT inhibited ZIKV replication in vitro, we evaluated the magnitude of the inhibition of virus replication in vivo. Therefore, we measured the viral load in the brain tissue on day 13 after infection. We observed that iDGAT treatment reduced mouse viremia by over threefold compared to untreated mice (Fig. 6B).

In addition, we evaluated the weight gain of the animals during the time course of the assay. ZIKV-infected animals stopped gaining weight from day 12 onward, whereas the weight gain of iDGAT-1-treated ZIKV-infected mice was indistinguishable compared to the animals from the control group (Fig. 6C). Most importantly, iDGAT-1-treated mice showed enhanced survival compared to nontreated mice (75% vs. 25%, respectively) (Fig. 6D).

Therefore, we observed that mice infected with ZIKV exhibited increased production of proinflammatory cytokines (TNF and IL-1β) and chemokines (CCL-2/MCP-1) in the brain tissue in comparison with mock-infected mice (Fig. 6E). Additionally, mice treated with the DGAT-1 inhibitor A922500 exhibited reduced inflammatory mediator production compared to nontreated mice during ZIKV infection. Our results showed that DGAT-1 inhibition reduced the ZIKV particle load and inflammatory profile and significantly protected against ZIKV-induced mortality in mice.

**Fig. 7:**
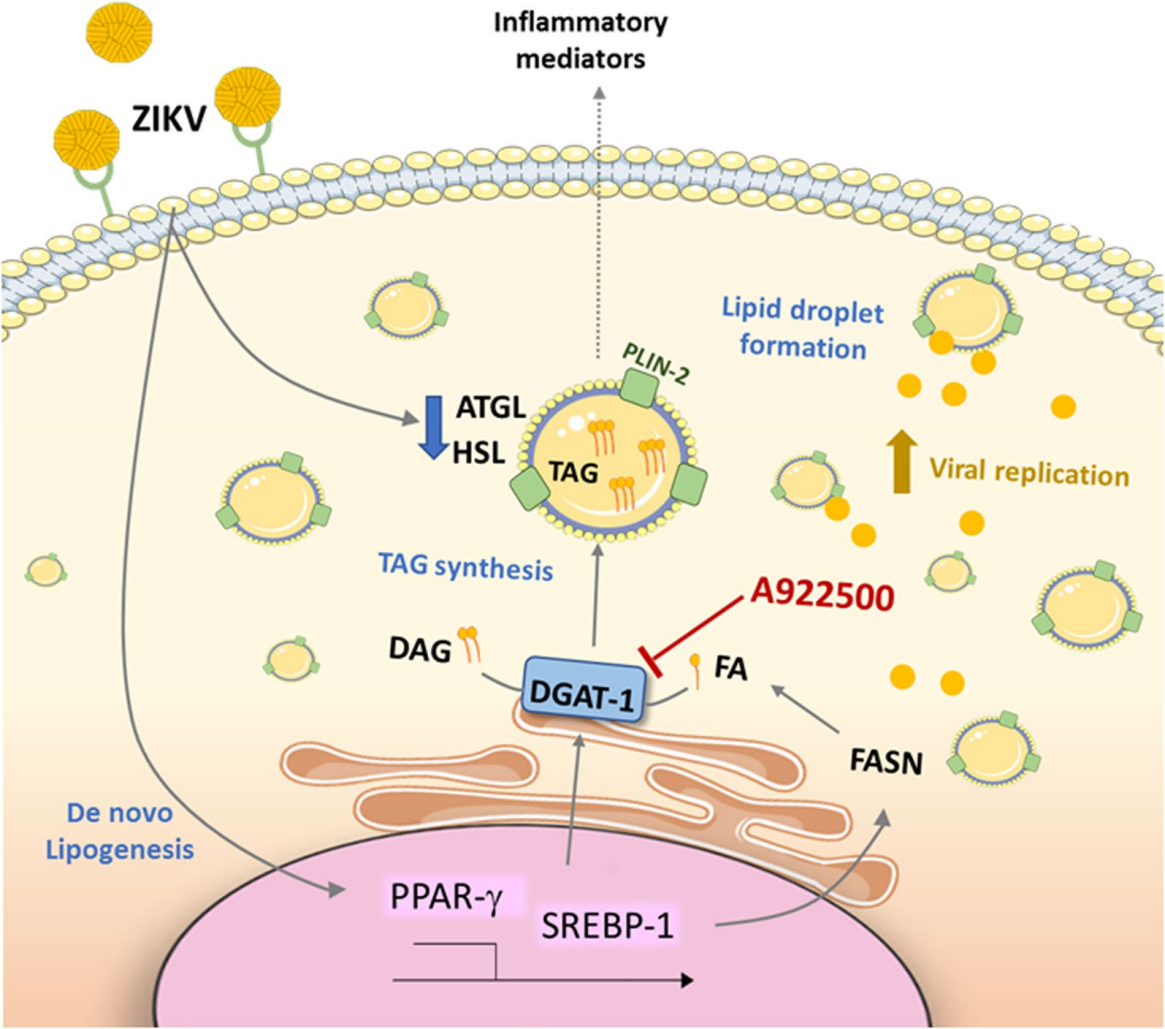
Conclusion. ZIKV is able to modulate the expression of important genes in lipid metabolism pathways, leading to increased levels of PPAR-γ and activation of SREBP-1. ZIKV infection increases the expression of FASN, which plays a role in fatty acid synthesis and is regulated by PPAR-γ. In addition, ZIKV decreases the levels of ATGL and HSL, important lipolytic enzymes. Therefore, these lipid metabolism regulations possibly contribute to the increase in LDs observed during ZIKV infection, and the biogenesis of this organelle is dependent on the DGAT-1 enzyme during infections. Treatment with the pharmacological inhibitor of DGAT-1, A922500, reduces the biogenesis of LDs and reduces the replication of ZIKV, contributing to the decrease in the production of inflammatory mediators. Altogether, our data suggest that LDs are important for the replication of ZIKV, participating in ZIKV pathogenesis

In summary, our results demonstrate that ZIKV modulates host lipid-related pathways leading to LD accumulation and suggest that this regulation (increased lipogenesis and decreased lipolysis) mainly depends on triglyceride metabolism. Therefore, the pharmacological targeting of LD formation to inhibit ZIKV replication is a potential strategy for antiviral development (Fig. 7).

## Discussion

The rapid spread of ZIKV, mainly in the Americas, led to a global alert state and intensified studies on this virus. However, to date, there is no specific treatment for ZIKV infection, and a complete cellular mechanism regulated by ZIKV to allow its replication in host cells is still unclear. Therefore, it is necessary to elucidate which dysfunctions are caused by the virus in neural cells, which somehow contribute to the pathogenesis of viral infection. Hence, in this work, we demonstrated that ZIKV upregulates lipogenesis pathways and downregulates lipolysis factors, leading to increased LD accumulation in human neural cells. In addition, the inhibition of DGAT-1 blocked LD biogenesis, reducing virus replication, inflammatory mediators and the viral load in the brain and improving mouse survival.

A variety of pathogens, including viruses, modulate cellular signaling and lipid metabolism to provide a more favorable environment for obtaining energy and replication [12, 31]; this feature has been well described in members of the family Flaviviridae, such as HCV and DENV [32, 45–47]. In this context, LD biogenesis plays a crucial role in maintaining intracellular energy storage and lipid homeostasis. Several enzymes, such as DGAT-1 and -2, tightly regulate the production of this organelle by controlling lipid synthesis [18]. These enzymes are essential in the biosynthesis of TAG, the main lipid constituent of LDs, and catalyze the final step in converting diacylglycerol (DAG) and fatty acids into TAG [48]. Lipolytic enzymes, such as HSL and ATGL, catabolize TAG stored in cellular LDs for lipid mobilization [49].

Our results demonstrate that ZIKV induces the expression and/or activation of transcription factors, such as PPAR-γ, SREBP-1 and FASN, and the production of LDs in human neural cells, suggesting cellular switching to a lipogenic phenotype. PPARγ is a transcription factor activated by lipid ligands and promotes multiple metabolic regulations, the expression of proteins involved in lipid homeostasis, and LD biogenesis [50, 51]. In consonance, the increase in this nuclear receptor has been associated with hepatic steatosis characterized by lipid accumulation during HCV infection [52, 53]. The activation of SREBP-1 occurs by the cleavage of the precursor SREBP-1 protein that converts it into the mature form, which regulates several metabolic genes acting on lipogenesis transcriptionally [54]. Accordingly, lipid accumulation induced by the HCV core in vitro depends on the activation of PPAR-γ and SREBP-1 [55, 56]. Indeed, inhibition of PPAR and SREBP-linked pathways is associated with HCV antiviral effects [57, 58].

Many stages of virus replication occur in the cytoplasm, altering host metabolism and interacting with structures to establish replication and assembly, as observed in LD organelles. Thus, Park et al. and Xiang et al. demonstrated that HCV nonstructural protein 4B (NS4B) [59] and nonstructural protein 5A (NS5A) induce SREBP-1 activation, contributing to lipid accumulation [60], respectively. Recently, it has been endorsed that AMP-activated protein kinase (AMPK) activation reverts hepatic lipid accumulation induced by the hepatitis virus [61]. In parallel, increased expression of FASN was also observed in virus-infected hepatocyte cell lines and mouse livers [59, 61, 62]. FASN is a key enzyme catalyzing the de novo synthesis of fatty acids and plays an essential role in lipogenesis. Moreover, recent results demonstrated lipid remodeling through SREBPs and increased FASN expression in human dendritic cells during ZIKV infection [63].

In addition to revealing significant expression of lipogenic factors induced by ZIKV, we observed that ZIKV decreases the expression of ATGL and HSL enzymes in neural cells. Under nutrient deprivation, for example, lipids are mobilized via the activation of lipolytic pathways. Perilipins found in LDs, which play a regulatory role in lipolysis, are degraded and allow the action of critical lipolytic enzymes such as ATGL and HSL involved in the intracellular degradation of TAGs [64]. Therefore, our results suggest that ZIKV fine-tunes LD biogenesis by tightening lipid metabolism pathways; the upregulation of transcription factors associated with lipogenesis and reduced lipolytic enzymes culminates in LD accumulation.

Furthermore, we identified that DGAT-1 expression increases after ZIKV infection; this enzyme catalyzes the last step in triglyceride synthesis. Interestingly, the DGAT-1 enzyme plays an essential role in the replication and assembly of the HCV virus since it allows the transport of the NS5 protein to the surface of the LDs [41]. Several strategies of inhibiting enzymes involved in lipid metabolism impact the biogenesis of LDs [14, 65]. The pharmacological inhibition of DGAT-1 reduced hepatic lipid accumulation and serum triglyceride concentrations in rodent models of postprandial hyperlipidemia [66, 67]. In the virus context, for instance, the pharmacological inhibitor of the DGAT-1 enzyme (A922500) reduced LD accumulation and decreased HCV replication [68]. Likewise, we demonstrated that ZIKV-induced DGAT-1 and the DGAT-1 inhibitor decreased the structural component of LDs, PLIN-2, which is necessary for the formation and maintenance of this organelle. Corroborating these observations, blocking DGAT-1 reduced the biogenesis of LDs induced by ZIKV infection and decreased viral replication in neural cells. Additionally, our results showed that oleic acid supplementation increased LD accumulation and ZIKV replication. These results are in agreement with recent findings demonstrating that oleic acid increased ZIKV replication in different cells [69]. Moreover, oleic acid treatment partially restored the inhibitory effect of A922500 on LD accumulation and ZIKV replication. Further supporting a role for LD on ZIKV replication in neural cells.

LDs are emerging as important organelles in the brain since LDs are present in different cells of the CNS under healthy and pathological conditions drawing attention to the potential functions of LDs during development, aging, and neurodegenerative diseases [26]. However, little is known about the regulation and functions of CNS LDs in the context of infection. To gain insight on the functions of LDs during brain infection, animals were pretreated with the DGAT-1 inhibitor prior to ZIKV infection. Indeed, the DGAT-1 inhibitor reduced not only the production of viral RNA, but also the proinflammatory mediators in mouse brains infected with ZIKV, such as TNF, IL-1β and MCP-1, suggesting an important role for CNS LDs in ZIKV replication in the brain and neuroinflammation. Moreover, our data revealed that treatment with a DGAT-1 inhibitor also decreased mouse mortality induced by ZIKV infection. These results indicate a role of the DGAT-1 enzyme and infection-induced LDs in ZIKV replication and neuropathology. Future studies will be necessary to characterize the involvement of LDs in other neuroinfections.

## Conclusion

ZIKV modulates the lipid metabolism of host cells by increasing the expression of important lipogenic proteins and decreasing lipolytic enzymes, contributing to a significant increase in LD accumulation in human neural cells. ZIKV-induced LD modulation provides a favorable environment for virus replication in cells susceptible to infection. Additionally, based on the data obtained, LDs participate in the ZIKV replicative cycle and neuroinflammation, and blocking DGAT-1 showed a protective effect in mice infected with ZIKV. Further studies are necessary to deeply understand the molecular mechanisms that regulate LD biogenesis and the contribution of this organelle to ZIKV pathogenesis. However, our results indicate that inhibiting DGAT-1 or other enzymes associated with lipid metabolism and LD accumulation could be a promising therapeutic strategy to control ZIKV.

## Materials and methods

### Cells and reagents

Human neuroblastoma cells (SH-SY5Y, ATCC CRL-2266) were cultured in Dulbecco's Modified Essential (DMEM) and F12 medium (GIBCO, supplemented with 10% fetal bovine serum (FSB, HyClone, Logan, Utah) and 100 U/mL penicillin‒streptomycin (P/S; GIBCO).

Human neural stem cells (NSCs) derived from iPS cells were prepared as previously described [2]. Human iPS cells were cultured in PSC neural induction medium (GIBCO, USA) containing neurobasal medium and PSC supplement for 7 days. Then, initial neural stem cells (NSCs) were split and expanded on neural induction medium (advanced DMEM/F12 and neurobasal medium (1:1) with neural induction supplement; Gibco).

African green monkey kidney (VERO subtype E6) cells were maintained in high glucose DMEM supplemented with 10% FBS and 100 U/mL P/S. All cell types were maintained at 37 °C in 5% CO_2_.

### Virus, infection, and titration

The ZIKV African (MR766) and Brazilian (GenBank accession #KX19720513) strains were propagated in VERO subtype E6 cells. These cells were infected at a multiplicity of infection (MOI) of 0.01 for 2 h at 37 °C. After that, the cells were cultured for 3 days in high glucose DMEM supplemented with 2% FBS. Then, cell lysates were obtained by freezing and thawing and centrifuged at 1,500 RPM at 4 °C for 5 min to remove cellular debris. Viral titers were quantified using the 50% tissue culture infectious dose (TCID50/mL) for further studies, and viruses were stored at − 70 °C. In parallel, cultures of noninfected VERO E6 cells were used as a mock control.

For experiments, SH-SY5Y cells were plated in DMEM/F-12 medium supplemented with 5% FSB and NSCs in Neurobasal, Advanced DMEM/F12, 2X NIS. Cells were infected 24 h after plating, upon reaching 80–90% confluency, using different MOIs of ZIKV and were incubated for 2 h for virus adsorption. After that, the virus inoculum medium was removed, and fresh medium was added. As controls, cells received only culture medium without FBS and VERO mock supernatants. In some experiments, cells were treated with a pharmacological inhibitor of the enzyme DGAT-1 (A922500—Sigma A11737) or dimethyl sulfoxide (DMSO—Sigma) as the vehicle control after infection.

Additionally, SH-SY5Y cells were pretreated or not with 40 μM oleic acid (Cat# O1008 –Sigma-Aldrich) in medium without FBS for 1 h prior to ZIKV infection. After ZIKV infection with an MOI of 1 for 2 h, the virus inoculum medium was removed, and fresh medium with 5% FSB was added with DGAT-1i (50 μM) or DMSO as the vehicle control in the presence or absence of oleic acid (40 μM) and analyzed after 48 h.

Viral titers were determined using the plaque-forming assay in VERO E6 cells seeded in 24-well plates. Cell monolayers were infected with different dilutions of the supernatant containing the virus in a tenfold serial dilution for one hour at 37 °C. After that, the cells were overlaid with high glucose DMEM containing 2% FBS and 2.4% carboxymethylcellulose, and after 3 days, they were fixed with 10% formaldehyde in PBS for 3 h. The cell monolayers were stained with 0.04% crystal violet in 20% ethanol for 1 h. The viral titer was calculated from the count of plaques formed in the wells corresponding to each dilution and expressed as plaque-forming units per mL (PFU/mL).

### Lipid droplet staining

SH-SY5Y cells were seeded on glass coverslips treated with 0.2℅ gelatin, and NSCs were plated on glass coverslips treated with Geltrex matrix (GIBCO) following the manufacturer's protocol. Cells were fixed with 3.7% formaldehyde, and LDs were stained with 0.3% Oil Red O (diluted in 60% isopropanol) for 2 min at room temperature. The coverslips were mounted on slides using antifade mounting medium (VECTASHIELD®). Nuclear recognition was based on DAPI staining (1 μg/mL) for 5 min. Fluorescence was analyzed by fluorescence microscopy with a 100 × objective lens (Olympus, Tokyo, Japan). The numbers of LDs were automatically quantified by ImageJ software analysis from 15 random fields.

### Immunofluorescence staining

SH-SY5Y cells were seeded in coverslips treated with 0.2% gelatin and, after 48 h, were fixed using 3.7% formaldehyde. Cells were rinsed three times with PBS containing 0.1 M CaCl_2_ and 1 M MgCl_2_ (PBS/CM) and then permeabilized with 0.1% Triton X-100 plus 0.2% BSA in PBS/CM for 10 min (PBS/CM/TB). The double-stranded RNA (ds-RNA) was labeled with mouse monoclonal antibody J2 clone—Scicons [54] at a 1:500 dilution overnight, followed by mouse anti-IgG-DyLight 550 or 488 at a 1:1000 dilution for one hour. LDs were stained with HCS LipidTOX™ Red Neutral Lipid Stain (Invitrogen) in PBS (Concentration 1:1000) for 30 min. The coverslips were mounted on slides using antifade mounting medium (VECTASHIELDs^®^). Nuclear recognition was based on DAPI staining (1 μg/mL) for 5 min. Fluorescence microscopy was analyzed with a 100 × objective lens (Olympus, Tokyo, Japan).

### SDS-PAGE and western blot

Forty-eight hours post-infection, cells were harvested using ice-cold lysis buffer pH 8.0 (1% Triton X-100, 2% SDS, 150 mM NaCl, 10 mM HEPES, 2 mM EDTA containing protease inhibitor cocktail—Roche). Cell lysates were heated at 100 °C for 5 min in Laemmli buffer pH 6.8 (20% β-mercaptoethanol; 370 mM Tris base; 160 μM bromophenol blue; 6% glycerol; 16% SDS). Thirty micrograms of protein/sample was resolved by electrophoresis on an SDS-containing 12% polyacrylamide gel (SDS-PAGE). After electrophoresis, the separated proteins were transferred to nitrocellulose membranes and incubated in blocking buffer (5% nonfat milk, 50 mM Tris–HCl, 150 mM NaCl, and 0.1% Tween 20). Membranes were probed overnight with the following antibodies: anti-PPARγ (Santa Cruz Biotechnology, #SC-7196-H100), anti-SREBP-1 (Abcam, Ab-28481), anti-HSL (Cell Signaling Technology, #4107), anti-ATGL (Santa Cruz Technology, # SC-365278), and anti-β-actin (Sigma, #A1978). After washing, they were incubated with IRDye—LICOR or HRP-conjugated secondary antibodies. All antibodies were diluted in blocking buffer. Signal detection was achieved with Supersignal Chemiluminescence (GE Healthcare) or fluorescence imaging using the Odyssey system. Densitometry was performed using Image Studio Lite Ver 5.2 software.

### Quantitative real-time RT-PCR assay

Twenty-four hours post-infection, the total RNA of SH-SY5Y monolayers was extracted and quantified by RT-PCR. The total RNA from each sample was extracted using the SV total RNA isolation system kit according to the manufacturer's protocols (Promega). RNA concentration and purity were determined by measuring absorbance at A260 and A280 nm with a Nanodrop 2000 spectrophotometer. RNA was stored at − 70 °C in nuclease-free water for later use. According to the manufacturer's protocol, 2 μg of total RNA was reverse transcribed in a 20-μl reaction mixture using the High Capacity cDNA Reverse Transcription kit (Applied Biosystems, Foster City, CA). The cDNA was amplified in 10 μl of 1 × TaqMan universal PCR master mix with Predeveloped TaqMan assay primers and probes Perilipin-2 (PLIN-2), Hs00605340_m1; diacylglycerol O-acyltransferase 1 (DGAT-1), Hs01020362_g1; fatty acid synthase (FASN) Hs01005622_m1 and beta-actin (ACTB) Hs99999903_m1 as endogenous controls (Thermo Fisher Scientific) according to the manufacturer's instructions. Quantitative RT-PCR was performed in a StepOne™ Real-Time PCR System (Thermo Fisher Scientific). PCR products were analyzed relative to the endogenous control ACTB (ΔΔCt).

### Cell viability assays

Cells were treated with a range of concentrations of A922500 for 48 h and fixed using 3.7% formaldehyde for 20 min. The cell monolayers were stained with 1% crystal violet in 20% ethanol for 10 min. Next, the cells were washed with water, and the crystal violet was extracted using methanol. The crystal violet was read in a spectrophotometer at a wavelength of 595 nm. Moreover, the cytotoxicity was determined according to the activity of lactate dehydrogenase (LDH) in the culture supernatants using a CytoTox® Kit according to the manufacturer’s instructions (Promega, USA).

### Animals

Swiss mice were supplied by the Institute of Science and Technology in Bio models (ICTB/Fiocruz) at approximately the 14th gestational day. The animals were kept at a constant temperature (25 °C) with free access to chow and water in a 12-h light/dark cycle. Pregnant mice were observed daily until delivery to determine the postnatal day accurately. We established a litter size of 10 animals for all experimental replicates. The Animal Welfare Committee of the Oswaldo Cruz Institute (CEUA/IOC) approved and covered the experiments in this study (license number L-001/2018). The procedures described in this study follow the local guidelines and guidelines published in the National Institutes of Health Guide for the Care and Use of Laboratory Animals.

### Experimental infection and treatment

Three-day-old Swiss mice were infected intraperitoneally with 2 × 10^4^ PFU of the Brazilian ZIKV strain. Treatments with iDGAT-1 were carried out at 2.5 mg/kg/day intraperitoneally and started one day before infection (pretreatment). The same dose was given once daily for the subsequent days throughout the experiment. For comparisons, a mock-infected group of animals was used as a control. Animals were monitored daily for survival and weight gain; euthanasia was performed to alleviate animal suffering when necessary. The criteria for humane endpoint were differences in weight between infected and control groups > 25%, ataxia or rejection of moribund offspring, characterized by no feeding by the female adult mouse.

### Measurements of inflammatory mediators in mouse brain homogenates

To evaluate the inflammatory process induced by ZIKV infection in the brain, mice were euthanized on day 13 after birth. Brains were perfused with 20 mL of PBS to remove the circulating blood and then collected, pottered, and homogenized in 500 µL of sterile PBS containing the complete EDTA-free protease inhibitor cocktail (Roche Applied Science, Mannheim, Germany) using an Ultra-Turrax Disperser T-10 basic IKA^®^ (Guangzhou, China). Homogenates were stored at − 80 °C for inflammatory mediator measurements. IL-1β, TNF-α and CCL2/MCP1 levels were quantified in brain extracts from infected mice by ELISA, following the manufacturer’s instructions (R&D Systems).

### Molecular detection of virus RNA levels

Brain tissue was collected on day 13 after birth. The brains were lysed with sterile 1 × PBS and homogenized with a homogenizer work center (IKA^®^ T10 basic). Homogenates were cleared by centrifugation, and total RNA was extracted. According to the manufacturer's instructions, total RNA from culture, extract-containing organs in 1 × PBS was extracted using QIAamp Viral RNA (Qiagen^®^). Quantitative RT-PCR was performed using the TaqMan^®^ Fast Virus 1-Step Master Mix kit (ThermoFisher^®^) in an ABI PRISM 7300 Sequence Detection System (Applied Biosystems). Amplifications were carried out in 10 µL reaction mixtures containing 2 × reaction mix buffer, 20 µM of each primer, 10 µM of the probe, and 5 µL of RNA template. Primers specific to ZIKV were used: Primer F (5′-CAG CTG GCA TCA AGA AYC-3′) and Primer R (5′-CAC YTG TCC CAT CTT YTT CTCC-3′). The standard curve method was employed for virus quantification. The Ct values for this target were compared for calibration, and the brain weight was used for normalization.

### Statistical analysis

Data are expressed as the mean ± standard error of the mean (SEM) of at least three independent experiments. The paired two-tailed t test was used to evaluate the significance of the comparison between two groups. Survival curves were evaluated using the log-rank (Mantel‒Cox) test. Multiple comparisons among three or more groups were performed with one-way ANOVA followed by Tukey's multiple comparison test using GraphPad Prism software 8.0. *P* values of 0.05 or less were considered statistically significant when comparing ZIKV infection to the uninfected control (*) group or ZIKV-infected and treated with A922500 group (#).

## Supplementary Information

**Additional file 1: Fig S1. **Treatment with A922500 decreases LD accumulation at 48 hpi in human neural cells. Representative images of (A) SH-SY5Y cells and (B) NSCs 48 h after ZIKV infection treated with a range of concentrations of the DGAT-1 inhibitor (A922500) and stained with Oil Red O (Red). The scale bar represents 20 µm in range. Data information: In (A-B), the data are presented as the means ± SEMs of three independent experiments. *P < 0.05 mock- versus ZIKV-infected cells. #P < 0.05 ZIKV-infected cells versus A922500 treatments.

**Additional file 2: Fig S2. **Cell cytotoxicity after A922500 treatment. Cells were treated with a range of concentrations of A922500 for 48 h. Cell viability using crystal violet staining of uninfected (A) SH-SY5Y cells and (B) NSCs treated with A922500. Cytotoxicity was evaluated by LDH activity in (C) SH-SY5Y cells and (D) NSCs. (E) CC_50_, EC_50_ and SI for SH-SY5Y cells and NSCs treated with A922500. Data information: In (A and C), the data are presented as the means ± SEMs of five independent experiments, and in (B and D), the data are presented as the means ± SEMs of four independent experiments. *P < 0.05 versus untreated cells.

## Data Availability

The datasets used and/or analyzed during the current study are included within the article or provided as supplementary materials.
