# Supplementary figures and images for: Metabolic reprogramming and lipid droplets are involved in Zika virus replication in neural cells
Source: J Neuroinflammation. 2023 Mar 8;20:61. doi: 10.1186/s12974-023-02736-7 (PMC9992922; doi:10.1186/s12974-023-02736-7)

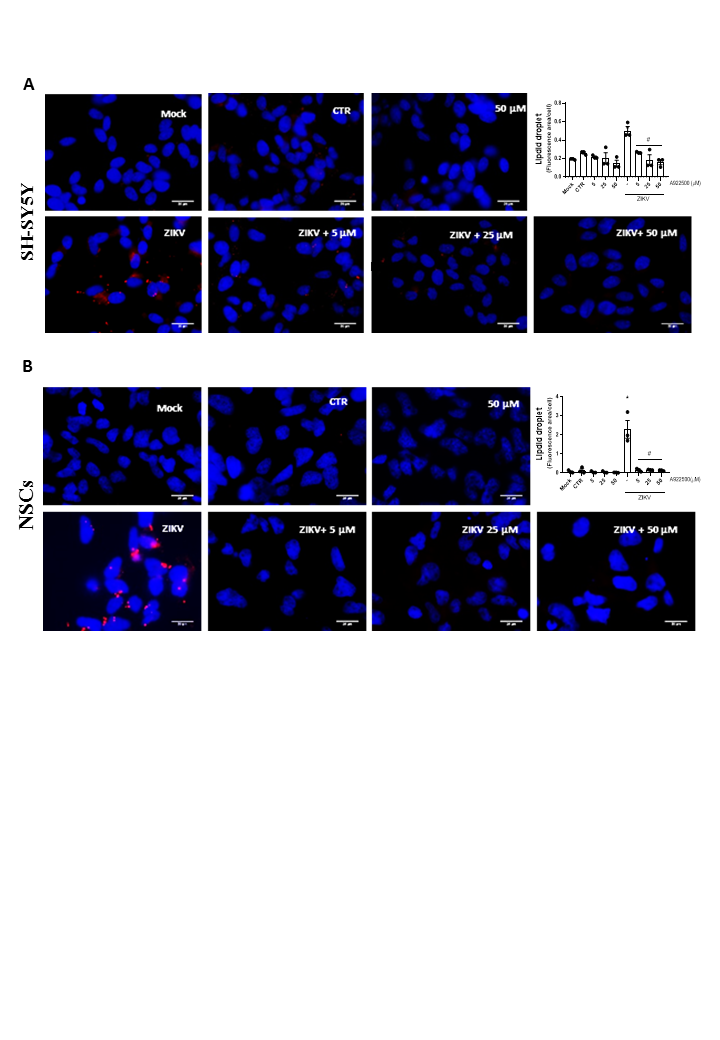

Supplement: Supplementary file 1 — Additional file 1: Fig S1. Treatment with A922500 decreases LD accumulation at 48 hpi in human neural cells. Representative images of (A) SH-SY5Y cells and (B) NSCs 48 h after ZIKV infection treated with a range of concentrations of the DGAT-1 inhibitor (A922500) and stained with Oil Red O (Red). The scale bar represents 20 µm in range. Data information: In (A-B), the data are presented as the means ± SEMs of three independent experiments. *P < 0.05 mock- versus ZIKV-infected cells. #P < 0.05 ZIKV-infected cells versus A922500 treatments. [file 12974_2023_2736_MOESM1_ESM.tif]

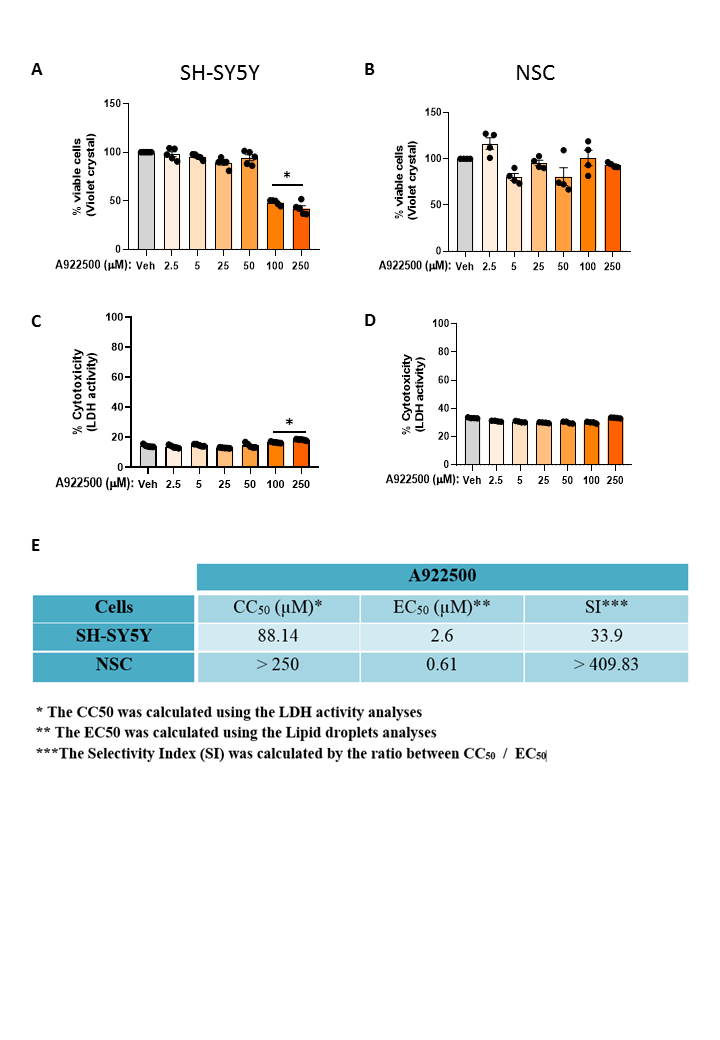

Supplement: Supplementary file 2 — Additional file 2: Fig S2. Cell cytotoxicity after A922500 treatment. Cells were treated with a range of concentrations of A922500 for 48 h. Cell viability using crystal violet staining of uninfected (A) SH-SY5Y cells and (B) NSCs treated with A922500. Cytotoxicity was evaluated by LDH activity in (C) SH-SY5Y cells and (D) NSCs. (E) CC50, EC50 and SI for SH-SY5Y cells and NSCs treated with A922500. Data information: In (A and C), the data are presented as the means ± SEMs of five independent experiments, and in (B and D), the data are presented as the means ± SEMs of four independent experiments. *P < 0.05 versus untreated cells. [file 12974_2023_2736_MOESM2_ESM.tif]
